# Informed consent: Old and new challenges in the context of the COVID-19 pandemic

**DOI:** 10.1017/cts.2021.401

**Published:** 2021-04-07

**Authors:** Erin Rothwell, Donna Brassil, Marietta Barton-Baxter, Kimberly A. Brownley, Neal W. Dickert, Daniel E. Ford, Stephanie A. Kraft, Jennifer B. McCormick, Benjamin S. Wilfond

**Affiliations:** 1 Department of Ob/Gyn, School of Medicine, University of Utah, Salt Lake City, UT, USA; 2 Rockefeller University, New York, NY, USA; 3 University of Kentucky Center for Clinical and Translational Science, Lexington, KY, USA; 4 Department of Psychiatry, School of Medicine, NC Translational and Clinical Sciences Institute, University of North Carolina at Chapel Hill, Chapel Hill, NC, USA; 5 Emory University School of Medicine, Department of Medicine, and Georgia Clinical and Translational Science Alliancet, Atlanta, GA, USA; 6 Johns Hopkins Institute for Clinical and Translational Research, Baltimore, MD, USA; 7 Treuman Katz Center for Pediatric Bioethics, Seattle Children’s Research Institute and Division of Bioethics and Palliative Care, Department of Pediatrics, University of Washington School of Medicine, Seattle, WA, USA; 8 Department of Humanities, College of Medicine, Pennsylvania State University, and Penn State Clinical and Translational Science Institute, Hershey, PA, USA; 9 Treuman Katz Center for Pediatric Bioethics, Seattle Children’s Hospital and Research Institute; Department of Pediatrics, University of Washington School of Medicine; Institute of Translational Health Sciences, Seattle, WA, USA

**Keywords:** Informed consent, COVID-19, pandemic, clinical research, CTSA

## Abstract

In this paper, we address how the COVID-19 pandemic has impacted informed consent for clinical research through examining experiences within Clinical and Translation Science Award (CTSA) institutions. We begin with a brief overview of informed consent and the challenges that existed prior to COVID-19. Then, we discuss how informed consent processes were modified or changed to address the pandemic, consider what lessons were learned, and present research and policy steps to prepare for future research and public health crises. The experiences and challenges for CTSA institutions offer an important perspective for examining what we have learned about informed consent and determining the next steps for improving the consent process.

## Informed Consent Practices Prior to the COVID-19 Pandemic

In recognition that the COVID-19 pandemic posed challenges related to informed consent or clinical research, the Food and Drug Administration (FDA) and Office for Human Research Protections (OHRP) both released statements related to human subjects protections in response to COVID-19 [1,2]. These guidance documents focused on details about electronic informed consent and reiterated that consent is more than a signature but a process [3]. The guidance documents were clear on the importance of comprehension, and protecting the rights and welfare of human subjects, and of recognizing that recruiting is the beginning of the consent process. These statements also reinforced aspirational goals of informed consent that have never been fully achieved.

The research literature provides substantial evidence about the inadequacies of the consent process for informed decision-making for research participation [4–17]. Some of the limitations that have been operative before and in the context of COVID-19 relate to the acuity of illness. For example, informed decision-making is difficult to facilitate when there is a need to make quick decisions to treat severe illness in the context of stressful situations and to communicate with surrogates about complex decisions they have not previously considered [18]. COVID-19 has also highlighted challenges related to coordination between research and clinical staff and to the need to provide interpreters for non-English-speaking participants [19,20]. And for many years, there have been prevalent concerns about comprehension in general with the growing complexity and length of consent documents [21–24].

Recent changes to the Common Rule (updated in 2017) reflected a recognition of some of these challenges. Specifically, there was an adoption of a new guiding principle for information disclosure and the requirement that informed consent forms begin with a concise summary of key information about the research study. Key information, importantly, is defined as the “information that is most likely to facilitate understanding of the reasons why one may or may not want to participate in research” [25]. This change reflects the understanding that in the decades since the Belmont Report was written, the quantity and complexity of information to be disclosed has increased, creating potential barriers to comprehension that could impede informed decisions about research participation. Much of the evolution of informed consent has been in response to comprehension challenges, and COVID-19 presents a new challenge in which consent continues to evolve.

To better understand the impact of the pandemic on long-standing concerns regarding informed consent in this setting, we conducted two group discussions with individuals (n = 15) from 15 Clinical and Translational Science Awards (CTSAs), IRBs, and other organizations involved in the protection of human participants. The discussions were intended to gather additional information about the experiences with informed consent in the context of COVID-19 from individuals at more CTSAs.

## Redefining, Modifying, and Streamlining Practices to Address the Challenges and Exigencies of the COVID-19 Pandemic

The challenges and exigencies of the COVID-19 pandemic described below were prior to the availability of vaccines, but nevertheless, provide critical lessons for improving informed consent in the future. Key challenges and changes with informed consent are listed in Table 1. The most obvious change to informed consent practice during the COVID-19 pandemic was that almost all of the procedures were conducted remotely and/or through electronic consent. (Table 2 outlines definitions and dimensions of these different modalities.) Not all institutions were able to offer e-consent, which caused some researchers to generate their own methods for obtaining consent and signatures for therapeutic trials. Even institutions that incorporated existing platforms, such as REDCap, for e-consent struggled to meet the increased demand from researchers for training and access to those platforms due to limited personnel to support this infrastructure. For studies that adopted e-consent platforms, issues involving the accessibility of the technology for potential participants and the lack of user-friendly interfaces to read the consent documents compounded the previous challenge of having complex, lengthy, and technical consent documents. Clearly, for gravely ill individuals and those with fatigue, the length of the informed consent document was a problem. Access to technology was even more difficult for underserved populations who were disproportionately impacted by COVID-19 [26].

**Table 1.** Challenges and changes with informed consent during COVID-19

Increased use of e-consent
Increased use of remote consent
Increase in barriers for obtaining signatures
Increased use of clinician team to consent
Increase in non-English-speaking participants
Increased use of legally authorized representatives
Increased use of waiver of signatures
Increase in re-consenting when capacity returned

**Table 2.** Consent approaches

Dimensions	Modalities	Classic^*^	E-consent^**^ (electronic information)	Remote^***^
Information	Written consent form only	+		
	Images, audio, or other multimedia components	+/−	+/−	+
Communication	In-person communication	+	+	
	Phone/video/email/text communication only		+/−	+
Documentation	Written signature	+		+
	Legal digital signature		+	+
	Check box only			+

* Classic Consent: Typically conducted in-person with a paper consent document and uses a written signature.

** Electronic information consent (e-consent): Refers to using electronic systems and processes that may employ multiple electronic media (e.g., text, graphics, audio, video, podcasts, and interactive web sites, biological recognition devices, and card readers) to convey information related to the study and to obtain and document informed consent.

*** Remote consent: Consent conducted without in-person contact and may include telephone, email, teleconference, text, and/or mail, but the signature is captured by the person signing the consent document or can be captured electronically.

The use of remote and/or e-consent added barriers to obtaining documentation of the participant’s signature when needed to be in compliance with Part 11 [27]. Essentially, for every COVID-19 treatment trial, the consent conversation posed an additional risk of infection to the study team member, and due to potential contamination, consent documents had to remain in the patient’s hospital room. These required changes to standard processes in order to avoid exposure for in-person consent went beyond the clinical care needed for standard treatment. Many of the institutions experienced a significant increase in requests to waive the need for a signature in the informed consent process, and requests were granted when the regulations permitted this exemption. Questions were raised about the importance of the signature when many of the methods for educating participants about the research study were verbal explanations because participants had difficulty reading the document.

Another notable challenge raised during COVID-19 was the limited availability of non-English-speaking staff to conduct the consent process and to provide translation of the consent documents. Institutions were not prepared to deal with the complexities of non-English-speaking participants. Some institutions used interpreters who normally only interpret during clinical care processes to obtain consent from non-English-speaking participants to address this need. Still, a lack of access to an adequate number of interpreters and a lack of resources to translate documents hindered the inclusion of participants from underrepresented minority groups in research trials. The quality of the interpretation and translation of documents was not assessed, and without quality assurance, may have resulted in poorer understanding compared to English-speaking participants [28].

The increased use of legally authorized representatives (LAR) was another challenge during COVID-19. One of the most common approaches was to discuss the study with the LAR over the phone and to document informed consent by having the LAR photograph the signed consent form and then email or text the picture to one of the study team members. This method had inherent challenges regarding initial communication about the study, answering participants’ questions, and obtaining the signature. Institutions also struggled to identify the best approaches to enable COVID-19 patients who had regained capacity and had been previously enrolled by a LAR in an acute care setting to re-consent. There were numerous situations where LAR was not willing or was unable to access a computer to review the consent document and sign it digitally. What was also apparent is that each LAR has different needs when it comes to informed consent: some want to speak with a study team member over the phone or through a video call while they walk through the paper document while others want to go through the consent document on their own.

The COVID-19 pandemic also highlighted the need for integration between research and clinical care into the consent process. Because of restrictions on direct patient contact, research staff had limited interactions with patients. Also, there were limited (at the beginning) therapeutic options for the treatment of COVID-19, and clinical trials represented the only option beyond supportive care for many patients. Further, the standard of care, and the presence of alternatives to participation in a given trial, represented a moving target that necessitated the involvement of clinical staff in helping patients to make informed decisions. Thus, the involvement of the clinical team was critical to consent, but we don’t know how it impacted willingness to participate and potential misperceptions of research versus clinical care.

## Key Lessons That Were Learned

Although much uncertainty remains, several key takeaways have emerged from the pandemic regarding key aspects of informed consent for research. Foremost, different approaches to informed consent can be executed that meet the FDA requirements and are acceptable for some of the population, although inaccessibility of the technology and inadequate capacity to communicate in languages other than English are significant barriers. The use of interpreters was helpful, and institutions will need to provide these services for more equitable access to research. Accessing consent materials from a computer may not be feasible or desirable for all participants; some people prefer to have someone call them and guide them through the consent process. Having multiple approaches helped to overcome these barriers for study teams. Researchers required additional training on how to develop consent forms beyond the paper-based approach and institutional resources should be increased to support these multiple options.

If lessons learned are to be actualized, the most important lesson learned is that institutions need to support translation and interpreters for more equitable and inclusive access to therapeutic research opportunities for the population in which the academic medical institution is serving. According to 21 CFR 50.20, the informed consent document should be in a language understandable to the subject (or authorized representative) [29]. Consent tools and processes that ignore non-English participants can no longer continue and it will be up to institutions and human research protection programs (HRPP) to address these important social justice issues. For example, clinical interpreters should be trained to interpret for research-informed consent, or alternatively, institutions should invest in interpreters specifically for research purposes. Having interpreters could greatly increase the availability of research opportunities to non-English speakers as well as increase personalization through human interaction.

Other concerns were already known but were highlighted and magnified by COVID-19. Specifically, more work is needed to understand how to better incorporate LARs into the decision-making process. Often, LARs were asked to make a decision about whether the patient would participate in research with limited knowledge about the current state of the patient’s health. Finally, the burden of comprehending a lengthy consent form during a time of concern for the patient in order for the patient/LAR to make an informed decision was also significant. The updated Common Rule provides some guidance on what a reasonable person should want to know and evaluating different formats and variations will be important for guiding future efforts to improve informed decision-making for research participation.

## Should the Novel Methods for Informed Consent Developed in Response to COVID-19 be Maintained or Discarded?

The pandemic spurred innovation and demonstrated the feasibility of different approaches to informed consent. Most concretely, e-consent and other remote consent methods were rapidly implemented in order to facilitate important research in a situation where traditional methods could not be used. Moving forward, more tools are now available to research teams, but important questions remain regarding how to use these tools most effectively in order to advance key goals of consent. It is especially important that novel platforms be harnessed to address, and not exacerbate, well-known problems with traditional informed consent. Challenges related to information overload remain, and the need to address what information is most important to communicate initially, during, and after participation in research, especially among those who are severely ill, should be addressed.

It is time to rethink what a reasonable person would want to know about a study in order to make an informed decision and how that information should be communicated such that it does not overly burden the participants and is accessible to individuals from a wide variety of communities. Prior to the pandemic, the typical informed consent process reflected historical norms and expectations specific to HRPPs and the federal regulations that were cemented in the 1980s-style approach utilizing face-to-face discussion, pen, and paper. Despite efforts to modernize and revolutionize the informed consent process, tradition has often prevailed, that is, until the pandemic made obtaining signatures in person from acutely ill COVID-19 patients unreasonably risky and virtually impossible. The pandemic forced HRPPs and researchers to fully acknowledge that informed consent is truly more than a signature on a form, and hopefully, it has resulted in enduring changes to the process that emphasize dialogue and prioritize voluntariness by using multiple, distinct methods and languages that flexibly meet the communication needs of research participants in a variety of contexts.

## Addressing Informed Consent in Future Pandemics or Public Health Crises

To learn from these experiences, an evaluation of research participants’ and families’ experiences with novel informed consent processes during COVID-19, using a mixed method, will help generate approaches to consent that promote a broader concept of respect for persons. Assessing different outcomes of the consent process beyond comprehensions such as trust and respect are equally important. Furthermore, if faced with a future pandemic, waiver of signature may want to be considered and the use of other forms of documentation. As described above, when dealing with acutely ill patients, the consent process was mostly verbal exchanges. A recording of a participant’s voice captured in an audio file might be sufficient, although possibly untenable, for acutely ill patients who are intubated. However, documentation by audio file captured via phone communication may help address technology (computer) barriers and facilitate the inclusion of the broader population in research studies.

Despite if another pandemic occurs, institutions should have some method for electronic consent available with means for rapid training for investigators, systematic assessment of whether the consent approach is acceptable for infected patients, and some alternative to electronic consent for those who do not find it accessible or acceptable. In addition, institutions and researchers should have plans in place for prioritizing specific studies and for informing participants about alternatives to research-based interventions. It is unknown how studies were prioritized during COVID-19 and the extent to which participation in other research studies was explicitly mentioned. Some have argued that all of the opportunities potential participants are eligible for should be presented in order for them to make an informed decision [30]. However, this is a significant burden and may not be possible for acutely ill patients. It is often clinicians who decide which trial is likely to be the most beneficial for their patients based on the therapeutic potential or for institutions to employ a randomization of the trials offered to patients. Institutions will need to consider how to make these decision-making processes transparent to potential participants.

The authors agree that COVID-19 has increased awareness among key stakeholders about the limits of informed consent and provides a unique opportunity for actionable change that can significantly improve access to research and informed decision-making for research participation. Overall, continual reevaluation of how the informed consent process should be conducted is essential. Institutional standards of practice for acceptable changes should be created and disseminated. Professional organizations and federal advisory groups may also want to identify research programs to improve the consent process.

Finally, it is important to acknowledge that informed consent has proven to be deceptively complex, and it has its limits. There are social, legal, and ethical challenges involved in informed decision-making for research participation. Respecting individuals’ autonomy may not be the only factor for consent. Others, such as the context of the situation (i.e., the urgency of treatment) and the right of the potential participant to make a decision with limited knowledge, can influence the consent process and individual autonomy. We cannot rely *only* on the informed consent process to communicate principles and values regarding the protection of individual rights and participant welfare. In other words, consent is important and, in many cases, necessary, but it is not sufficient to ensure respect and appropriate protection. Research is needed to identify novel mechanisms for upholding the ethical principles of respect for persons and additional functions of consent beyond comprehension. For all of these issues, it is important that study teams, institutions, and HRPPs work together in partnership. The COVID-19 pandemic prompted investigators and HRPPs to embrace new ways of approaching consent, and it has energized efforts to rethink the consent experience and determine how to better communicate with potential study participants. A potential research agenda for investigating informed consent practices and policies could consider the following suggestions:

1. Put more emphasis on the process than the document. This has been said numerous times, but if we can develop better communication and decision support tools that are participant-centered and focus on the relationship rather than disclosure this may help. Some research has identified that participants already make the decision to participate in research before being asked [31]. Is this because they have a relationship with the institutions? What about individuals who do not have a previous relationship and/or do not trust the institutions? Clearly adding more information to the consent document is not the solution.
2. Explore alternative mechanisms for communicating information beyond reading the text. The use of visual images and verbal exchanges may be more effective for promoting informed decision-making. Institutions need to provide resources for investigators to develop quality consent tools that promote understanding and address literacy concerns as well as trainings for recruiters for cultural competency and implicit bias.
3. Consider adding to the one-time consent encounter follow-up communication. During COVID-19, re-consent was obtained from patients after they regained capacity, but adding follow-up communication to all participants in general to promote not only more understanding, but also transparency and participant centeredness is important.
4. Updates to the Common Rule highlight the importance of providing a concise summary of the information that a “reasonable person” would want to know. How do we consider what information is necessary to communicate? COVID-19 has shown ways that operationalizing can be difficult, both from content and method. Community engagement with real-world consent forms to identify examples of what is considered most important could help guide investigators.
5. Institutions need to provide resources for consent translation and interpreters. The lack of these resources significantly hindered recruitment and may harm by denying inclusive access to therapeutic interventions. Clinical interpreters and integration with the clinical team were critical to the consent process during COVID-19. The preconceived practice to separate research outside the clinical encounter changed, but research is needed to identify if this integration only served to further blur the distinction between what is research and what is clinical, or did it improve more equitable access to research?
